# Consistent metagenomic biomarker detection via robust PCA

**DOI:** 10.1186/s13062-017-0175-4

**Published:** 2017-01-31

**Authors:** Mustafa Alshawaqfeh, Ahmad Bashaireh, Erchin Serpedin, Jan Suchodolski

**Affiliations:** 1 Bioinformatics and Genomic Signal Processing Lab, ECEN Dept., Texas A&M University, College Station, 77843-3128 TX USA; 2 College of Veterinary Medicine and Biomedical Sciences, Gastrointestinal Laboratory, Texas A&M University, College Station, 77843-3128 TX USA

**Keywords:** Biomarker detection, Metagenomics, Robust PCA

## Abstract

**Background:**

Recent developments of high throughput sequencing technologies allow the characterization of the microbial communities inhabiting our world. Various metagenomic studies have suggested using microbial taxa as potential biomarkers for certain diseases. In practice, the number of available samples varies from experiment to experiment. Therefore, a robust biomarker detection algorithm is needed to provide a set of potential markers irrespective of the number of available samples. Consistent performance is essential to derive solid biological conclusions and to transfer these findings into clinical applications. Surprisingly, the consistency of a metagenomic biomarker detection algorithm with respect to the variation in the experiment size has not been addressed by the current state-of-art algorithms.

**Results:**

We propose a consistency-classification framework that enables the assessment of consistency and classification performance of a biomarker discovery algorithm. This evaluation protocol is based on random resampling to mimic the variation in the experiment size. Moreover, we model the metagenomic data matrix as a superposition of two matrices. The first matrix is a low-rank matrix that models the abundance levels of the irrelevant bacteria. The second matrix is a sparse matrix that captures the abundance levels of the bacteria that are differentially abundant between different phenotypes. Then, we propose a novel Robust Principal Component Analysis (RPCA) based biomarker discovery algorithm to recover the sparse matrix. RPCA belongs to the class of multivariate feature selection methods which treat the features collectively rather than individually. This provides the proposed algorithm with an inherent ability to handle the complex microbial interactions. Comprehensive comparisons of RPCA with the state-of-the-art algorithms on two realistic datasets are conducted. Results show that RPCA consistently outperforms the other algorithms in terms of classification accuracy and reproducibility performance.

**Conclusions:**

The RPCA-based biomarker detection algorithm provides a high reproducibility performance irrespective of the complexity of the dataset or the number of selected biomarkers. Also, RPCA selects biomarkers with quite high discriminative accuracy. Thus, RPCA is a consistent and accurate tool for selecting taxanomical biomarkers for different microbial populations.

**Reviewers:**

This article was reviewed by Masanori Arita and Zoltan Gaspari.

**Electronic supplementary material:**

The online version of this article (doi:10.1186/s13062-017-0175-4) contains supplementary material, which is available to authorized users.

## Background

With trillions of microbes inhabiting the human body, bacteria play an essential role in defining the health and disease states of the host. In general, these microbial inhabitants outnumber the human’s cells and comprise about 150 times more genes than the human genome [1]. Some studies have reported that the microbes outnumber the human’s cells by a ratio of 10:1 [2], while others limits this ratio to 1.3:1 [3]. However, investigating the bacterial communities has been limited in the past because more than 90% of microbes are unknown and uncultivable [4, 5]. Recent advancements in sequencing technologies have overcome these limitations and provided researchers with the taxonomic composition and functional capacity of microbial colonies [6, 7].

Various metagenomic studies have associated the imbalance in bacterial communities with certain diseases ranging from obesity [8–10], diabetes [11], inflammatory bowel disease (IBD) [12] to cancer [13, 14]. This suggests using microbes as potential biomarkers for host’s health and disease states. Moreover, the continuous development of inexpensive, high throughput sequencing technologies has prompted the widespread use of microbial biomarker discovery studies.

Biomarker detection presents itself as a major means of translating metagenomic data into clinical practice [15]. Identifying potential biomarkers is essential in understanding disease evolution and designing antibiotic and/or probiotic therapies. Microbial biomarker discovery aims to identify the specific operational taxonomic units (OTUs), whose relative abundances differ between different phenotypes. Mathematically, identifying biomarkers is cast as the problem of finding the most informative variables (or features) that discriminate two or more groups of samples (i.e., healthy versus diseased, or different disease stages).

Identifying the most discriminating features in metagenomic datasets is a challenging task for several reasons. First, the number of features representing potential biomarkers is large, a challenge that is commonly referred to as ‘the curse of dimensionality’. The challenges associated with analyzing the high dimensional metagenomic data set is compounded by the small number of available samples. This high-dimension small-sample challenge raises serious analytical challenges [16, 17]. Second, many microbial populations exhibit a high inter-subject variability. For example, [9] shows that the gut bacterial ecosystems of twins differ significantly. This inter-subject variability adds more confounding factors that complicate the analysis and interpretation of the results. Third, the microbial communities exhibit a high dynamics due to the complex relationships between its members [18–20] and the direct interaction with the host [21]. Fourth, metagenomic data are subject to their own artifacts including sequencing errors and chimeric reads [22, 23].

Ensuring the reproducibility of the results drawn from biological data is crucial for clinical applications and to prevent incorrect biological conclusions. For example, around 70 gene markers for breast cancer were identified by each of the two large-scale gene expression studies [24, 25]. However, only three genes were found to be common between the two sets of identified biomarkers [26]. As an additional example, in the work of Ressom et al. [27], only seven features have been consistently appeared in the best 7–8 detected biomarkers determined using a combination of particle swarm optimization (PSO) and support vector machine (SVM) as well as in the 128 detected biomarkers detected via a filtering approach based on t-test. These facts point out a serious inconsistency problem that prevents many biomarker detection algorithms from identifying the correct biomarkers involved in the biological process under study. Therefore, measuring the *consistency* of biomarkers discovery algorithms is an important metric in the assessment and design of such algorithms.

The biomarker discovery problem can be tackled in two general frameworks: (*i*) the statistical framework, and (*ii*) the machine learning framework. In general, the statistical methods resume to applying a statistical test to compute a p-value for each feature and selecting the features with p-values lower than a predefined threshold as biomarkers. The associated multiple hypothesis problems are typically handled by replacing the *p*-values with false discovery rates (FDRs). Currently, Metastats [28] and LEFSe [15] are the only two available methods that explicitly apply statistical assessment approach of metagenomic differences for metagenomic biomarker discovery. Metastats employs a nonparametric t-test using permutations for the non-sparse features and exact feature tests for the sparse features. LEFSe couples the statistical analysis with the effect size estimation to achieve a robust biomarker discovery. For statistical assessment, LEFSe employs the non-parametric Kruskal-Wallis and Wilcoxon-Mann–Whitney tests for class and subclass comparisons, respectively.

From the machine learning perspective, the biomarker detection task is cast as a feature selection problem. The most commonly used feature selection methods are the filtering approaches. In filtering methods, each feature (i.e., OTU) is scored individually and independently of the other features. Then, the OTUs with high scores are selected as potential biomarkers. The score for each feature is calculated according to the relevance between the abundance levels of the OTU in the samples and the class labels of the samples. The individual and independent ranking renders the filtering methods to be computationally simple and fast. On the other hand, individual ranking ignores the dependencies between variables. In other words, individual and independent ranking in filtering methods neglects the fact that a feature may be irrelevant individually but strongly relevant if it is combined with other features.

To overcome the individual scoring problem, the feature transformation approaches attempt to adopt more informative features where each new feature is a function of all the initial features. Hence, the dependencies between features are implicitly expressed in the constructed features. Depending on whether the class labels are considered in the transform function, the feature transformation methods are broadly divided into supervised and unsupervised methods. The most prominent unsupervised method is the principal component analysis (PCA). PCA employs an orthogonal linear transformation that seeks preserving the variance of the data. In the category of supervised methods, partial least squares (PLS) and linear discriminant analysis (LDA) are the widely used methods. These methods have been extensively used for the analysis of biological data.

Recently, the authors in [29] have proposed an algorithm to recover the low-rank and sparse matrices from their superposition. This matrix recovery problem is referred to as robust PCA (RPCA). It can be easily described by means of the following matrix decomposition. Assume matrix **D** is decomposed as follows:

1 $$ \mathbf{D} = \mathbf{L} + \mathbf{S},   $$

where **L** is a low-rank matrix and **S** is a sparse perturbation matrix. The entries of **L** are of arbitrary magnitude and its low-dimensional column and row spaces are unknown. Similarly, the number and location of the nonzero entries of **S** are unknown and they can assume arbitrary large magnitudes. The key feature of RPCA is that it can recover both **L** and **S** by solving a convex optimization problem, referred to as Principal Component Pursuit.

Surprisingly, the two state-of-the-art biomarker detection algorithms (i.e., MetaStats and LEFSe) have not included the stability performance in the assessment of the quality of the detected biomarkers. Therefore, we propose a protocol for evaluating a biomarker detection algorithm in terms of both (i) the consistency of the detected biomarkers and (ii) the classification performance. This proposed protocol was motivated by the model selection approach developed in [30] to find the optimal feature selection-classifier combination for a given dataset. Moreover, we propose a novel method to identify microbial biomarkers based on RPCA. The essence of our proposed method is to model the differentially and non-differentially abundant OTUs as a sparse and low-rank matrix, respectively. The reasoning behind this model lies in the fact that the majority of the microbes are irrelevant to the biological process at hand. Therefore, these irrelevant OTUs are supposed to have abundance levels that do not vary between two different phenotypes (i.e., healthy and diseased). Hence, it is natural to consider their abundance level matrix as a low-rank matrix (denoted by **L**). On the other hand, the abundance levels of the few relevant OTUs exhibit significant variations between the two phenotypes. This can be represented by a sparse matrix (denoted by **S**). The RPCA is employed to decompose the OTUs abundances matrix into the superposition of **L** and **S**. Then, the bacterial biomarkers are identified based on the recovered matrix **S**. Although the RPCA exhibited success in several applications such as surveillance video and face recognition [29], and identifying differentially expressed genes from gene expression data [31], it has not been applied to identify potential bacterial markers from metagenomic data. Moreover, the consistency performance of RPCA has not been addressed in [31].

## Methods

### Data description

Unless stated otherwise, the 16S rRNA gene sequencing reads were assigned to operational taxonomic units (OTUs) using the naive Bayesian classifier employed by the Ribosomal Database Project (RDP) [32]. Reads with confidence below 80% were assigned to be uncertain. For all the datasets described below, the per-sample normalized read counts were organized in a matrix called the taxonomic relative abundances matrix. This matrix is the final input for the RPCA algorithm. As RPCA belongs to the unsupervised family of machine learning algorithms, the labels of the data are not required.

#### Canine inflammatory bowel disease (IBD) dataset

Naturally passed fecal samples were obtained from 89 healthy dogs and 79 dogs with chronic signs of gastrointestinal disease and confirmed inflammatory changes on histopathology. All dogs participated in different clinical studies and leftover fecal samples were utilized for this study.

Dogs with clinical signs of chronic GI disease (i.e., vomiting, diarrhea, anorexia, weight loss, etc.) were diagnosed with idiopathic IBD based on the World Small Animal Veterinary Association (WSAVA) criteria: (i) chronic (i.e., ≥ 3 weeks) GI signs; (ii) histopathologic evidence of mucosal inflammation; (iii) inability to document other causes of GI inflammation; (iv) inadequate response to dietary, antibiotic, and anthelmintic therapies, and (v) clinical response to anti-inflammatory or immunosuppressive agents. Histological samples were obtained endoscopically. Clinical status of each dog was evaluated using a published clinical canine IBD activity index (CIBDAI). Within the IBD dogs, 47 dogs had histological confirmed inflammation in the small intestine, 24 dogs had histological changes in both small intestine and colon, and 7 dogs had only histological changes reported in the colon. Histological changes were predominantly of lymphoplasmacytic infiltrates, with a subset of dogs also showing eosinophilic and/or neutrophilic components. Data can be downloaded from this link: https://qiita.ucsd.edu/study/description/833.

#### Mouse model of ulcerative colitis (UC) dataset

This dataset represents the fecal microbiota of mice model with ulcerative colitis and control mice. In particular, the microbiota of 20 T-bet ^−/−^ x Rag2 ^−/−^ (UC) and 10 Rag2 ^−/−^ (control) mice was characterized using 16S data from fecal samples. The data is publicly available in the supplementary material of [15].

### Consistency-classification evaluation protocol

The proposed protocol is based on measuring the consistency and the classification performance over different variations of the original dataset. In particular, an empirical estimation of the consistency has been designed based on the idea that a stable biomarker detection algorithm should yield similar results under small variations of the dataset. This complies with the expectations of biologists that modifying the original dataset by adding or removing a few samples should not lead to a significant change on the identified biomarkers by an algorithm. Consequently, the procedure for estimating the consistency assumes the following steps. The first step is to repeatedly, for K times, subsample the original dataset $\mathbf {D} \in \Re _{+}^{p\times N}$ into two subsets: $\mathbf {D}_{k}^{train} \in \Re _{+}^{p\times \left \lceil rN\right \rceil }$ and $\mathbf {D}_{k}^{test} \in \Re _{+}^{p\times (N-\left \lceil rN\right \rceil)}$, where *k* stands for the iteration number. The second step is to apply the biomarker detection algorithm on the $\left \{\mathbf {D}_{k}^{train} \right \}_{k=1}^{K}$ subsets to find *K* sets of potential markers. The third step is to measure the pairwise similarity between the $\tfrac {K(K-1)}{2}$ pairs of the biomarker sets using a similarity or stability index. Then, the overall consistency (*C*_*avg*_) of the algorithm is defined as the average of all pairwise similarities. Mathematically,

2 $$ C_{{avg}} = \frac{2\sum_{i=1}^{K}{\sum_{j=i+1}^{K}{SI(\mathcal{F}_{i},\mathcal{F}_{j})}}}{K(K-1)},   $$

where $\mathcal {F}_{i}$ denotes the output of the biomarker detection method over the *i* ^′^ *th* subsample. $SI(\mathcal {F}_{i},\mathcal {F}_{j})$ represents the similarity between two marker sets measured by the stability (i.e., similarity) index *SI*.

**Fig. 1:**
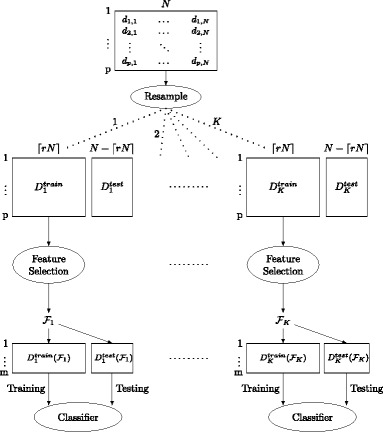
Consistency-classification evaluation protocol

Similarly, we use the same subsamples to evaluate the classification performance. Particularly, the data corresponding to the selected markers in each generated training and testing subsets are extracted and are denoted by $\mathbf {D}_{k}^{train}(\mathcal {F}_{k})$ and $\mathbf {D}_{k}^{test}(\mathcal {F}_{k})$, respectively. The $\mathbf {D}_{k}^{train}(\mathcal {F}_{k})$ subset is utilized to train the classifier, while the $\mathbf {D}_{k}^{test}(\mathcal {F}_{k})$ serves as an independent set for testing the classifier. Repeating the evaluation for *K* times reduces the risk of over-optimistic results of the conventional cross-validation on small-sample studies [33]. This consistency-classification evaluation protocol is summarized in Fig 1.

#### Consistency performance

Several measures have been proposed to measure the similarity between two sets (i.e., the output of a biomarker detection algorithm over two subsamples). In this work, we adopt the Kuncheva index (KI) [34] as a measure of similarity. KI is defined as

3 $$ KI(\mathcal{F}_{i},\mathcal{F}_{j}) = \frac{p.|\mathcal{F}_{i} \cap \mathcal{F}_{j}| - T^{2}}{T.(p - T)} = \frac{|\mathcal{F}_{i} \cap \mathcal{F}_{j}| - (T^{2}/p)}{T - (T^{2}/p)},   $$

where $T = |\mathcal {F}_{i}| = |\mathcal {F}_{j}|$. The Kuncheva index ranges from −1 to 1. The larger the value, the more common biomarkers among the two sets $\mathcal {F}_{i}$ and $ \mathcal {F}_{j}$. Negative values indicate that the shared biomarkers are mostly due to chance. Negative values can be obtained due to the correction term (*T* ^2^/*p*) that aims to compensate for possible bias due to the randomly selected biomarkers and are common among the two marker lists.

#### Classification performance

The classification performance is measured in terms of sensitivity, specificity and accuracy. The accuracy represents the portion of the correctly classified instances in both classes (ex., healthy and diseased). In case of imbalanced class distribution, accuracy becomes misleading since it is dominated by the majority class. This is particularly true when the prediction of the minority group is critical. Therefore, to complete the picture about the classification performance, class-specific measures such as sensitivity and specificity are also important. Sensitivity and specificity are defined as the portion of correctly predicted instances in the positive (i.e., diseased) and negative (i.e., healthy) classes, respectively. Let *TN* and *TP* denote the number of correctly identified negative and positive samples, respectively. Also, let *FN* and *FP* represent the number of false-classified samples in the negative and positive classes, respectively. Then, the accuracy, sensitivity and specificity are defined as follows:

4 $$\begin{array}{*{20}l} Accuracy &= \frac{TP+TN}{TP+FN+TN+FP},  \end{array} $$

5 $$\begin{array}{*{20}l} Sensitivity &= \frac{TP}{TP+FN},  \end{array} $$

6 $$\begin{array}{*{20}l} Specificity &= \frac{TN}{TN+FP}.  \end{array} $$

### Robust principal component analysis

RPCA is a matrix recovery problem which aims to recover the low-rank matrix **L** and the sparse matrix **S** from their superposition **D**. The authors in [29, 35] have shown that under broad assumptions, it is possible to *exactly* recover both components (i.e., low rank and sparse matrices) by solving a convex optimization problem called *Principal Component Pursuit (PCP)*. PCP aims to minimize a weighted sum of the nuclear norm of the low-rank matrix and of the *l*_1_ norm of the sparse matrix. Mathematically, PCP is expressed as

7 $$\begin{array}{*{20}l} &\text{minimize} ~~ \|\mathbf{L}\|_{*} + \lambda \|\mathbf{S}\|_{1} \\ &\textrm{subject to} ~~ \mathbf{D} = \mathbf{L} + \mathbf{S},  \end{array} $$

where *λ* is a positive regularization parameter that controls the sparseness and smoothness of **S** and **L**, respectively. ∥**L**∥_∗_ denotes the nuclear norm of the matrix **L** and it is equal to the sum of the singular values of the matrix. ∥**S**∥_1_ represents the *l*_1_ norm of the matrix and it is equal to the sum of the absolute values of all the matrix entries.

Various methods have been proposed for solving the PCP problem such as the iterative thresholding approach [36] and the accelerated proximal gradient approach [37]. In this paper, we adopt the augmented Lagrange multiplier (ALM) algorithm to solve (7). In general, ALM algorithms solve constrained optimization problems by converting them into unconstrained problems with a new objective called the *augmented Lagrangian*. The *augmented Lagrangian* for the PCP problem is given by

8 $$ \begin{array}{ll} l(\mathbf{L},\mathbf{S},\mathbf{Y}) &= \|\mathbf{L}\|_{*} + \lambda\|\mathbf{S}\|_{1} + \\ & \left\langle \mathbf{Y},\mathbf{D}-\mathbf{L}-\mathbf{S}\right\rangle + \frac{\mu}{2}\|\mathbf{D}-\mathbf{L}-\mathbf{S}\|_{F}^{2}, \end{array}   $$

where **Y** represents the Lagrange multiplier matrix, and *μ* stands for the single regularization parameter associated with the ALM formulation. Thus, the ALM formulation of the PCP problem is given by

9 $$ \begin{array}{ll} \text{minimize} ~~ & l(\mathbf{L},\mathbf{S},\mathbf{Y}) = \|\mathbf{L}\|_{*} + \lambda\|\mathbf{S}\|_{1} +\\ & \left\langle \mathbf{Y},\mathbf{D}-\mathbf{L}-\mathbf{S}\right\rangle + \frac{\mu}{2}\|\mathbf{D}-\mathbf{L}-\mathbf{S}\|_{F}^{2}. \end{array}   $$

A standard approach to solve (9) is of iterative-based nature. Each iteration *k* consists of two steps. The first step is to solve the following sub-problem

10 $$ (\mathbf{L}_{k}^{*},\mathbf{S}_{k}^{*}) = \text{arg}\min_{\mathbf{L},\mathbf{S}} ~~ l(\mathbf{L},\mathbf{S},\mathbf{Y}_{k}).   $$

The second step is to update the Lagrange multiplier matrix using the following equation

11 $$ \mathbf{Y}_{k+1} = \mathbf{Y}_{k} + \mu \left(\mathbf{D}-\mathbf{L}_{k}-\mathbf{S}_{k}\right).   $$

Since a jointly optimal solution for the sub-problem (10) is not available, a practical and efficient solution is to employ the alternating optimization algorithm. This alternating-based method first minimizes *l*(**L**,**S**,**Y**_*k*_) with respect to **L** (**S** is fixed), then it minimizes *l*(**L**,**S**,**Y**_*k*_) with respect to **S** (**L** is fixed). This strategy utilizes the fact that both *min*_**L**_{*l*(**L**,**S**,**Y**)} and *min*_**S**_{*l*(**L**,**S**,**Y**)} have a closed form solution. In particular, let $\mathcal {S}_{\tau }:\Re \to \Re $ be the shrinkage operator defined by

12 $$ \mathcal{S}_{\tau}(x) = \text{sgn}(x) \text{max}(|x|-\tau,0),   $$

where *τ*≥0 represents the threshold value. This shrinkage operator is extended to matrices by applying it to their elements. Then,

13 $$ \begin{array}{ll} \mathbf{S}^{*} &= \text{arg}\min_{\mathbf{S}} ~~ l(\mathbf{L},\mathbf{S},\mathbf{Y}) \\ & = \mathcal{S}_{\lambda \mu^{-1}}(\mathbf{D}-\mathbf{L}+\mu^{-1} \mathbf{Y}). \end{array}   $$

To solve for **L**, let $\mathcal {D}_{\tau }$ denotes the singular value thresholding operator given by

14 $$ \mathcal{D}_{\tau}(\mathbf{X}) = \mathbf{U} \mathcal{S}_{\tau}(\Sigma)\mathbf{V}^{T},   $$

where **X**=**U** *Σ* **V** ^*T*^ is the singular value decomposition (SVD) of **X**. Then,

15 $$ \begin{array}{ll} \mathbf{L}^{*} &= \text{arg}\min_{\mathbf{L}} ~~ l(\mathbf{L},\mathbf{S},\mathbf{Y}) \\ & = \mathcal{D}_{\mu^{-1}}(\mathbf{D}-\mathbf{S}+\mu^{-1} \mathbf{Y}). \end{array}   $$

Though we have a closed-form solution for $\mathbf {S}_{k}^{*}$ and $\mathbf {L}_{k}^{*}$, solving the sub-problem (10) requires computing (13) and (15) repeatedly until converging to the optimal solution. This repetition leads to a significant computation burden. According to [35], this burden can be avoided by updating **S**_*k*_ and **L**_*k*_ only once. Even though this does not guarantee the optimal solution of the sub-problem (10), it is sufficient to converge to the optimal solution of the RPCA problem as proved in [35].

### Extracting the differentially abundant bacteria via RPCA

The proposed method for identifying metagenomic biomarkers is divided into two steps. First, apply RPCA to decompose the original bacterial abundance level data into a low-rank matrix representing the non-differential abundant bacteria and a sparse matrix representing the differential abundant bacteria. Second, score each microbe (i.e., feature) by constructing a scoring vector based on the extracted sparse matrix. The top *m* bacteria are selected as biomarkers for the biological process under study.

Consider the bacterial abundance level matrix $\mathbf {D} \in \Re _{+}^{p\times N}$. Each column of **D** (denoted by **d**_*i*_) represents the abundance levels of the *p* microbes in one sample. Each row represents the abundance level of one bacteria in all the *N* samples. Typically, *p*≫*N* which signifies a classical high-dimensional small-sample problem. As mentioned in the Introduction section, it is reasonable to consider the observed abundance matrix **D** as being the sum between a low-rank matrix **L** and a sparse matrix **S**. Potential biomarkers are expected to exhibit abundance levels that vary between samples belonging to different groups. Therefore, their abundance levels can be modeled as a sparse perturbation matrix superimposed over the low-rank matrix representing the abundance levels of the non-differentiable microbes (i.e., **D**=**L**+**S**). Consequently, the microbial biomarkers can be detected according to the sparse matrix **S**. The bacteria exhibiting more variation are stronger. The extracted sparse matrix **S** can be expressed as

16 $$ \mathbf{S} = \left[\begin{array}{llll} s_{11} & s_{12} & \hdots &s_{1n} \\ s_{21} & s_{22} & \hdots &s_{2n} \\ \vdots & \vdots & \ddots &\vdots \\ s_{p1} & s_{p2} & \hdots &s_{{pn}} \end{array}\right] = [\mathbf{s}_{1}, \mathbf{s}_{2}, \hdots, \mathbf{s}_{n}].   $$

Corresponding to the original abundance level data matrix **D**, each column contains the differential abundance levels of all the microbes in one sample, and each row of **S** represents the differential variation of a microbe in all the *N* samples. The entries of **S** can be either positive or negative reflecting whether the bacteria were activated or deactivated in response to the biological process. Therefore, the absolute values of the entries in **S** are needed for the identification of the differentially abundant bacteria (i.e., biomarkers). The score of the *i* ^′^ *th* bacteria is calculated by summing row wise the absolute values of the *i* ^′^ *th* row in **S**. Mathematically, the scoring vector (**v**) is obtained by summing the absolute values of the elements of **S**, and can be expressed as:

17 $$ \mathbf{v} = \left[\sum_{j=1}^{N}|s_{1j}|,\hdots,\sum_{j=1}^{N}|s_{{pj}}|\right]^{T}.   $$

Large scores are associated with microbes exhibiting larger variation between the two states. Therefore, only the genes with the top *r* scores are selected as biomarkers.

### Nearest centroid classifier (NCC)

A nearest centroid classifier is an instance of distance-based supervised learning method. The classification process using NCC consists of two steps. The first step is to train the classifier with labeled data (i.e., **d**_*i*_) to compute the mean (i.e., centroid) of each class. The mean of the *k* ^′^ *th* class ($\mathbf {\mu }_{C_{k}}$) is defined by:

18 $$ \mathbf{\mu}_{C_{k}} = \frac{1}{|N_{C_{k}}|}\sum_{\mathbf{d}_{i} \in C_{k}}\mathbf{d}_{i},   $$

where $|N_{C_{k}}|$ denotes the number of samples belonging to *k* ^′^ *th* class. The second step reduces to assigning a test sample (**z**) to the class whose centroid is closer. Mathematically, this is equivalent to the following optimization problem:

19 $$ \hat{C}(\mathbf{z}) = \text{arg}\min_{C_{k}}dis(\mu_{C_{k}},\mathbf{z})   $$

where $dis(\mu _{C_{k}},\mathbf {z})$ is a distance measure between the test sample **z** and the centroid of the *k* ^′^ *th* classifier ($\mathbf {\mu }_{C_{k}}$).

## Results and discussions

This section presents the experimental evaluations on the two metagenomic studies described in the Material and Methods section. The performance of our proposed scheme, RPCA, is compared with the current state-of-the-art algorithms proposed for identifying microbial biomarkers. In particular, RPCA is compared with two statistical-based algorithms namely, MetaStats [28] and LEFSe [15], and two machine learning-based algorithms. For the machine learning-based algorithms, an entropy-based and a binary classification (BC)-based [38] filtering approach are used.

The five algorithms were evaluated in terms of their classification and consistency performance according to the consistency-classification evaluation protocol shown in Fig. 1. In our experiments, 500 subsamples (i.e., *K*=500) were generated by randomly subsampling, without replacement, the original datasets. Due to the limited number of samples in metagenomic studies, subsamples were generated with 80*%* of the samples in the original dataset (i.e., *r*=0.8). The reported results represent the average over the 500 experiments.

To reduce the dependency of the results on the classification criteria, two variants of the nearest centroid classifiers were used. In the first approach, the *l*_1_ norm was used as a measure of distance, while in the second approach, the *l*_2_ norm was used. In this paper, we refer to the first classifier as NCC-1 and to the second one as NCC-2. The consistency of the biomarker detection algorithms has been measured by the Kuncheva index. In order to study the impact of the number of selected features on the consistency and classification performance, the five biomarker detection algorithms were assessed at different sizes of the biomarker sets.

It is worth to mention that there are several implementations for the RPCA algorithm. In our experiments, we utilize the Matlab code for the exact ALM provided by the authors of [37], which is available at ‘http://perception.csl.illinois.edu/matrix-rank/sample_code.html’.

### Canine inflammatory bowel disease (IBD) dataset

**Fig. 2:**
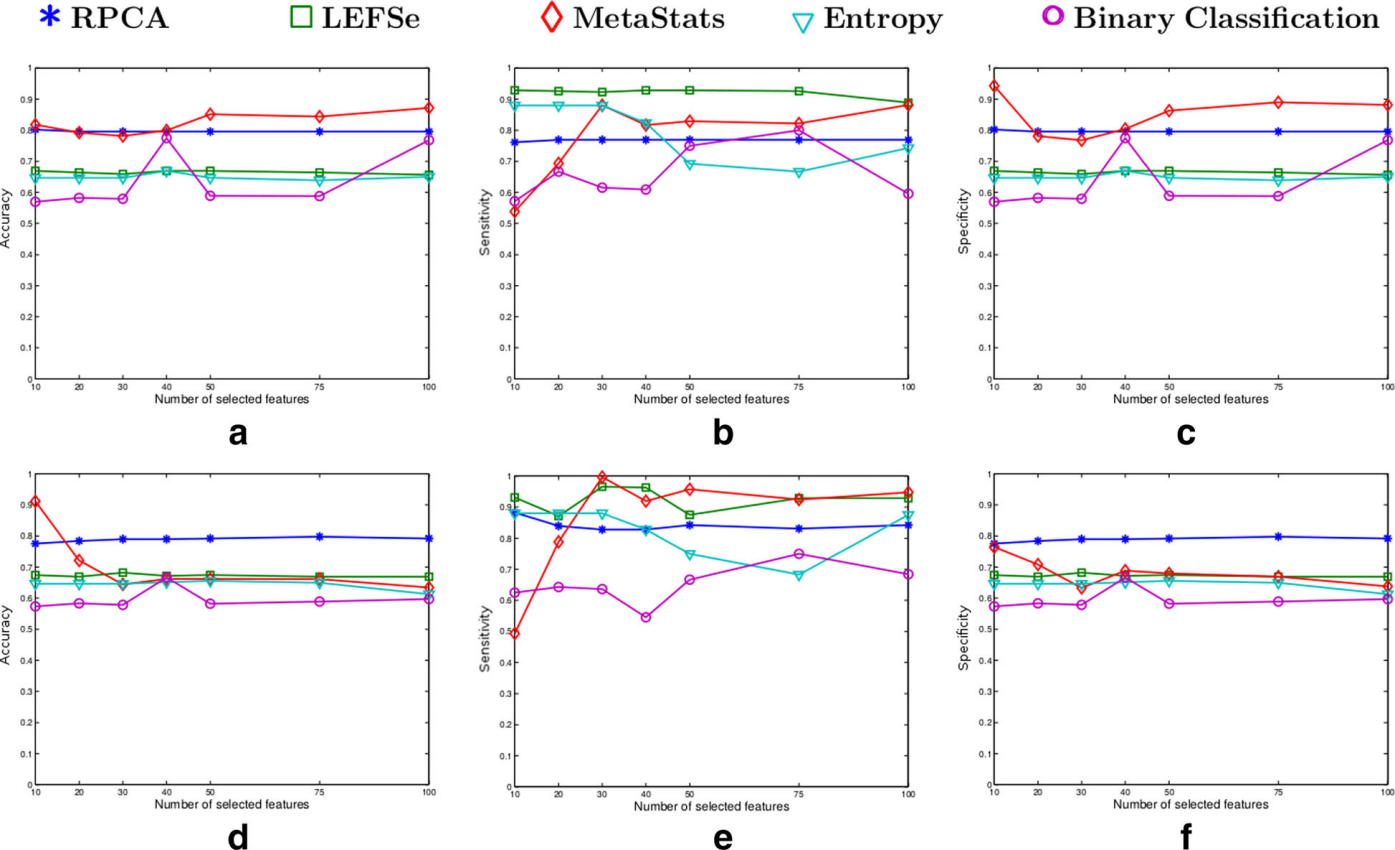
Classification performance of the five algorithms over the canine IBD dataset in terms of accuracy, sensitivity and specificity. The first row represents the results corresponding to the NCC-1 classifier (**a** accuracy, **b** sensitivity, **c** specificity), while the second row represents the NCC-2 classifier results (**d** accuracy, **e** sensitivity, **f** specificity)

The performance of the five algorithms in terms of their classification accuracy for varying number of biomarkers from the canine IBD dataset is depicted in Fig. 2. The first row in Fig. 2 presents the results for the NCC-1 classifier, while the second row presents the results for the NCC-2 classifier. As the results displayed in Figs.2a and b illustrate, RPCA outperforms the LEFSe, entropy-based and BC-based algorithms in terms of accuracy. In particular, the RPCA algorithm outperforms the LEFSe and entropy-based algorithms by around 13% and the BC-based algorithm by approximately 20%. The MetaStats algorithm provides comparable results to RPCA when NCC-1 is used. However, the RPBC algorithm significantly outperforms the MetaStats performance in the NCC-2 case. Moreover, RPCA provides a robust result irrespective of the variation in the applied classification method and the number of selected biomarkers. This contrasts the performance of MetaStats.

**Fig. 3:**
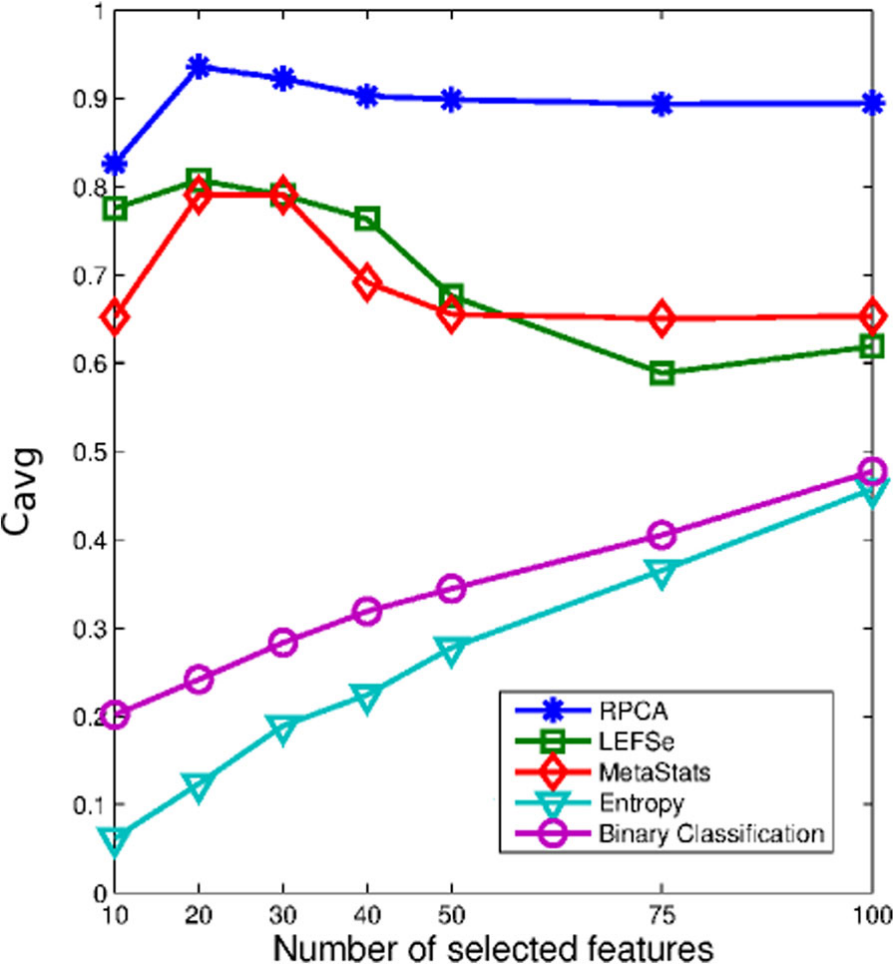
The average consistency performance measured by KI of the five biomarker discovery algorithms over the canine IBD dataset

**Fig. 4:**
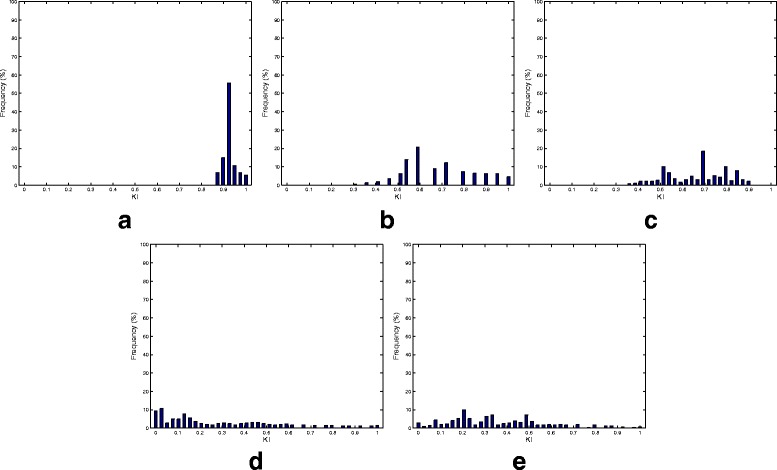
Histogram plots of all the 124750 pairwise KIs (i.e., $\tfrac {K(K-1)}{2}= 124750$ comparisons; *K*=500) generated by the five biomarker discovery algorithms over the canine IBD dataset. **a** RPCA. **b** LEFSe. **c** MetaStats. **d** Entropy. **e** Binary classification

Our next simulation sought to examine the consistency performance of the five methods. Figure 3 presents the KI stability values averaged over all the pairwise comparisons (i.e., $\frac {K(K-1)}{2}= 124750$ comparisons; *K*=500). In addition to the superior consistency performance, RPCA shows a robust performance irrespective of the number of selected markers. Detailed consistency analysis when the size of the selected biomarkers equals 50 is depicted in Fig. 4 by presenting the histogram of the KI index computed over all pairwise comparisons. As is turns out from Fig. 4 a, the RPCA algorithm shows a high consistent performance. This is revealed form the concentration of the histogram corresponding to RPCA at high consistency values. In particular, for almost 80% of the times, RPCA provides a stability value that is larger than or equal to 90%. On the other hand, LEFSe and MetaStats turn out to present inconsistent performance. For example, LEFSe presents KI values less than or equal to 60% for almost half of the times. The entropy-based and BC-based algorithms yield a very poor consistency performance.

**Fig. 5:**
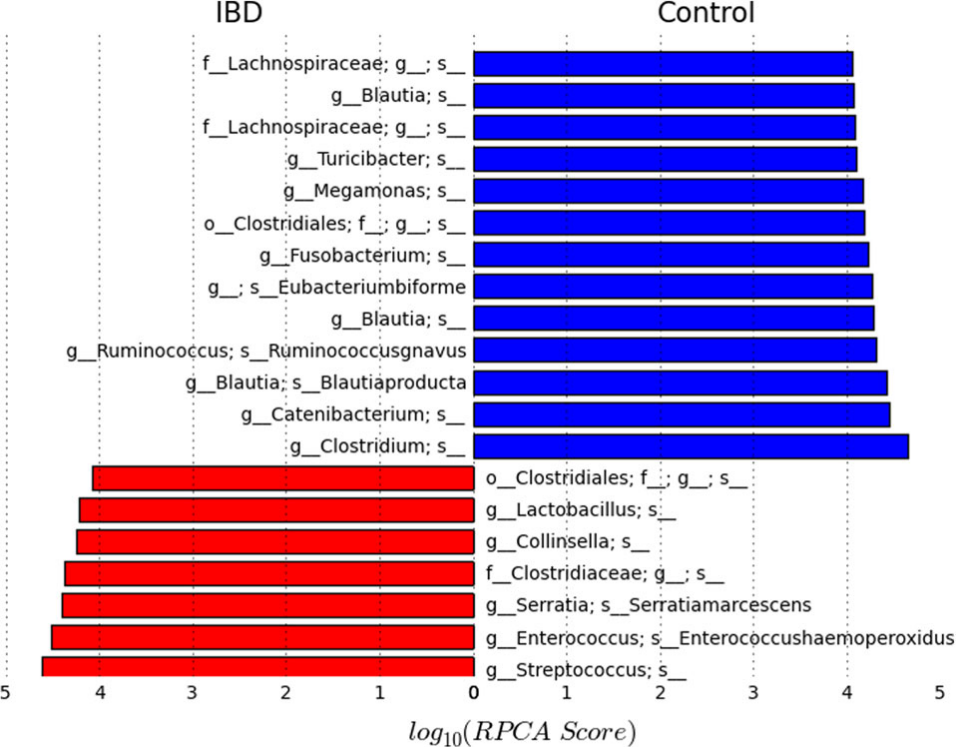
Top 20 identified biomarkers by RPCA in relation to the canine IBD dataset and their RPCA scores. *Blue*: the selected bacteria exhibit an increase in their abundance level in the control samples. *Red*: the selected bacteria exhibit an increase in their abundance level in the IBD samples

The top 20 detected biomarkers by the RPCA algorithm are shown with their scores in Fig. 5. According to [39], Erysipelotrichaceae is considered to be a major player in maintaining homeostasis in response to inflammation. This may explain the selection of two clades (i.e., Eubacteriumbiforme and g_Catenibacterium) that belong to the Erysipelotrichaceae family as possible biomarkers for IBD. In agreement with previous studies, Collinsella [40] shows an increase in its abundance, whereas Turicibacter [41] exhibits reduced concentration in IBD subjects. This may explain selecting species belonging to these clades as potential biomarker for IBD.

Species belong to several genera, including Blautia (i.e., Blautiaproducta and two unspecified species), Ruminococcus (i.e., Ruminococcusgnavus), and a number of taxa within the family of Lachnospiraceae show decreased abundances in IBD patients [42]. On the other hand, Lactobacillus and Streptococcus exhibit an increase in their abundance levels in patients with Crohn’s disease [42]. Fusobacterium has previously been suggested as a biomarker for IBD [43].

In order to validate the detected markers by RPCA, an independent validation experiment has been conducted. In particular, quantitative PCR (qPCR) assays targeting thirteen bacterial groups were conducted over fecal DNA samples taken from 285 healthy dogs and 172 dogs with chronic enteropathy (CE) [44]. In this experiment, the final PCR panel (i.e., Faecalibacterium, Turicibacter, E. coli, Streptococcus, Blautia, and Fusobacterium) includes five OTUs (i.e., Faecalibacterium, Turicibacter, Streptococcus, Blautia, and Fusobacterium) that are strongly suggested as potential signature for IBD by our RPCA-based algorithm.

As shown in the Additional file 1, the RPCA is the only algorithm that has suggested Faecalibacterium and Blautia as potential biomarker for IBD. In particular, Blautia has been proposed as a strong driver for IBD by RPCA and three species from Blautia genera (rank 5, 9, and 18) were selected in the top 30 markers. Moreover, the Streptococcus has been strongly suggested as strong potential marker for IBD by RPCA. Specifically, one species of Streptococcus was ranked second by RPCA. This agrees with Metastats which suggested two species belonging to Streptococcus genera (rank 3 and 8), and BC which suggested one Streptococcus species with rank 23 as IBD marker. On the other hand LEFSe and Entropy algorithms do not include Streptococcus in their suggested lists of IBD markers. RPCA and LEFSe are the only algorithms that suggested Turicibacter as a signature for IBD. Fusobacterium was strongly recommended as possible marker for IBD by RPCA and MetaStats (rank 12 and 5, respectively) while it is less favored by LEFSe (rank 25). This independent validation experiment demonstrates the efficiency of RPCA-based algorithm in identifying markers with high classification potential.

### Mouse model of ulcerative colitis (UC) dataset

**Fig. 6:**
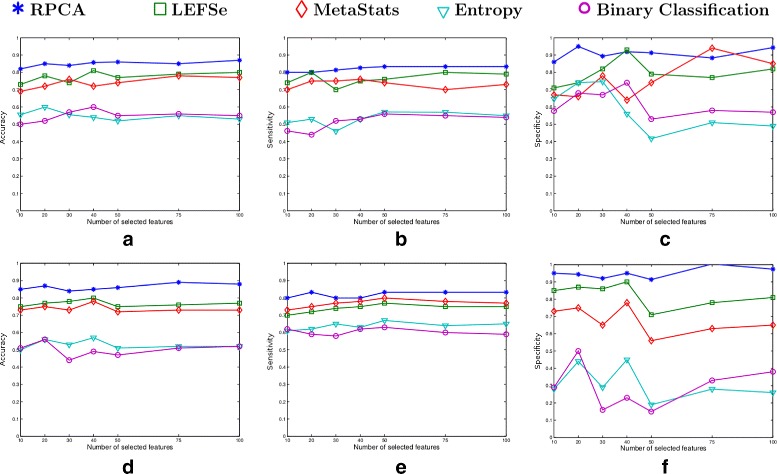
Classification performance of the five algorithms over the mouse model of UC dataset in terms of accuracy, sensitivity and specificity. The first row represents the results corresponding to the NCC-1 classifier (**a** accuracy, **b** sensitivity, **c** specificity), while the second row represents the NCC-2 classifier results (**d** accuracy, **e** sensitivity, **f** specificity)

Figure 6 presents the classification performance of the five algorithms for varying number of biomarkers from the ulcerative colitis mice model dataset. The first and second row represents the classification performance corresponding to the NCC-1 and NCC-2 classifier, respectively.

The results in Fig. 6 a and d demonstrate that the RPCA algorithm outperforms all the four methods in terms of classification accuracy. Moreover, RPCA exhibits a consistent performance regardless of the classification method and the number of biomarkers included in the classifier models. On the other hand, the other four algorithms exhibit a variation in their accuracy by around 10% when varying the number of selected markers from 10 to 100.

**Fig. 7:**
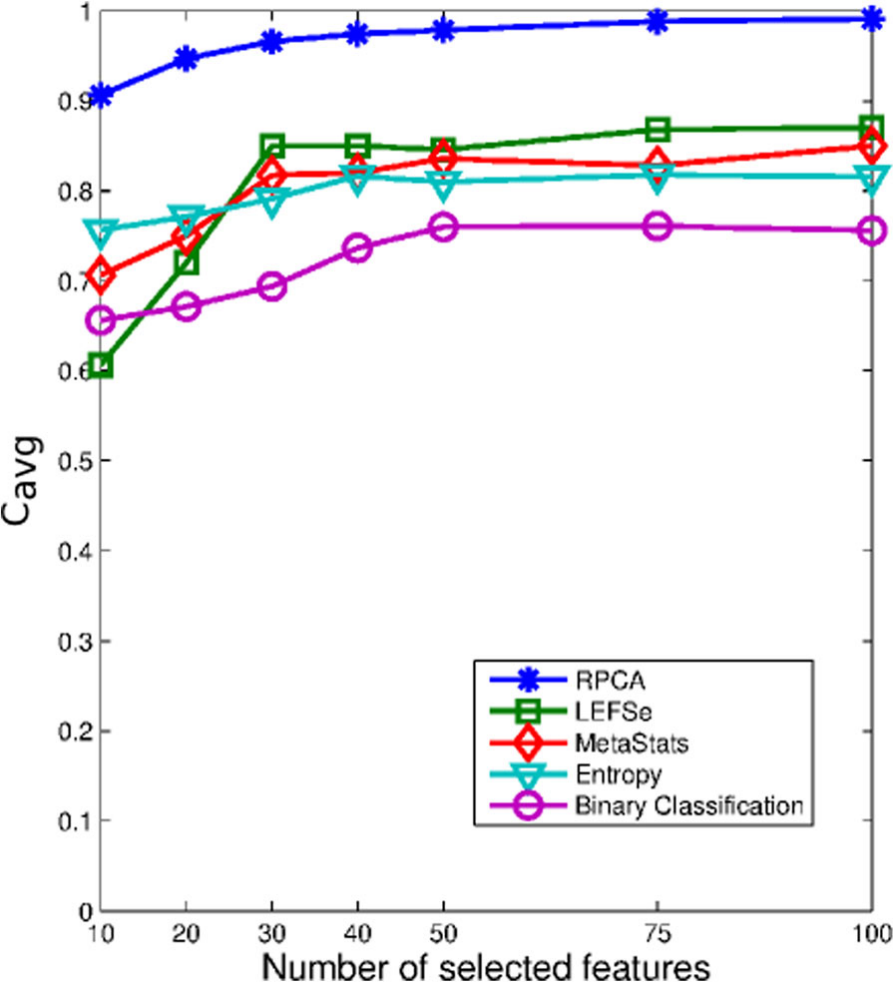
The average consistency performance measured by KI of the five biomarker discovery algorithms over the mouse model of UC dataset

**Fig. 8:**
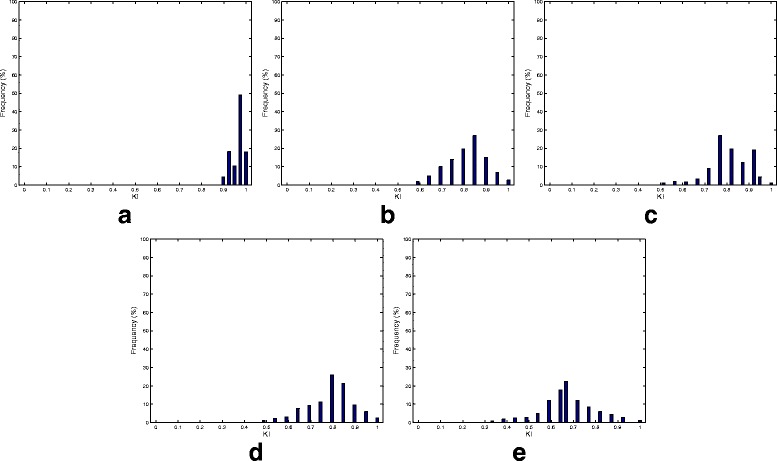
Histogram plots of all the 124750 pairwise KIs (i.e., $\tfrac {K(K-1)}{2}= 124750$ comparisons; *K*=500) generated by the five biomarker discovery algorithms over the mouse model of UC dataset. **a** RPCA. **b** LEFSe. **c** MetaStats. **d** Entropy. **e** Binary classification

The average KI values over all the pairwise comparisons and their histogram when the number of selected biomarkers equals 30 are depicted in Figs. 7 and 8, respectively. Figure 7 points out that RPCA exhibits a very high consistency performance and outperforms all the other algorithms. In particular, when the number of markers is larger than 30, RPCA provides an improvement by around 10–15% over the LEFSe, MetaStats and entropy-based algorithms, and approximately 20% over the BC-based method. This improvement increases when the number of markers is less than 30. For example, for a marker set of size 10, this gain increases to 30 and 20% when RPCA is compared to MetaStats and LEFSe, respectively.

The distributions of the all pairwise KI for the five algorithms are depicted in Fig. 8. These histograms provide a finer view of the consistency performance of the algorithms. Figure 8 a demonstrates that the RPCA algorithm provides a consistent performance as the KI values exceeds 95% for almost 80% of the times. The other algorithms show much less consistent performance compared to RPCA. This behavior is clear from the facts that the histograms of these methods are centered at lower values for KI and the wide spread of KI values.

**Fig. 9:**
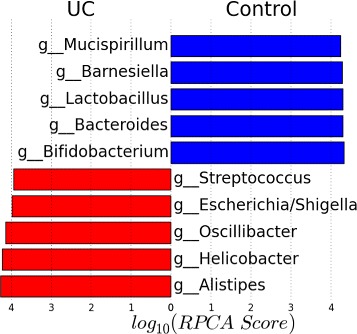
Top 10 identified biomarkers by RPCA in relation to the mouse model of ulcerative colitis dataset and their RPCA scores. *Blue*: the selected bacteria exhibit an increase in their abundance level in control samples. *Red*: the selected bacteria exhibit an increase in their abundance level in UC samples

The top 10 identified biomarkers by the RPCA algorithm are listed in Fig. 9. RPCA suggests the enrichment of Oscillibacter, Alistipes, Helicobacter and Escherichia/Shigella as potential biomarkers for UC. This agrees with previous studies. For example, the authors of [45] found that Alistipes presents a very low abundance level in almost all patients diagnosed with UC. The previous study [12] reported that consistent reductions of acetate producer clades such as Ruminococcaceae, to which Oscillibacter belongs, may negatively impact the host ability to repair the epithelium and to regulate inflammation. For Helicobacter, the authors of [46] reported significant lower rates of Helicobacter pylori, the most widely known species of Helicobacter genus, in UC patients. Also, the increased levels of the Escherichia/Shigella has been linked to the intestinal inflammation [47].

In agreement with the previous studies, RPCA associates the reduction in the concentration of Lactobacillus, Bifidobacterium and Bacteroides to UC. Previous studies have reported similar results. For example, decreased concentrations of Lactobacillus and Bifidobacterium in colonic biopsy specimens and reduced faecal concentrations of lactobacilli and bifidobacteria have been found in patients with active UC [48, 49]. According to [50], the UC can be characterized by the decrease in the abundance levels of Bacteroides. This analysis highlights the agreement of RPCA with the biological findings and suggests additional taxa as possible biomarkers for UC.

## Conclusions

Recent advancements in metagenomic sequencing enable researchers to investigate the diverse microbial communities with reasonable costs. Despite these advancements, analyzing these massive metagenomic datasets to derive consistent biological conclusions is a challenge. Due to the increasing number of metagenomic studies that associate microbes with several diseases, identifying potential metagenomic biomarkers in relation to a specific biological process is crucial for (a) understanding that process, and (b) developing possible therapies. Therefore, robust and consistent analytical techniques that ensure the reproducibility of the results are essential for clinical applications. Toward this end, we first described an evaluation protocol that assesses the reproducibility performance of the biomarker detection algorithm, as shown in Fig. 1. This evaluation protocol estimates the consistency (i.e., reproducibility) performance based on generating different variations of the original data using random subsampling without replacement. Then, a biomarker detection algorithm is employed over all the generated subsamples. The pairwise similarity between all the marker lists is computed using a measure of similarity. Second, we proposed a RPCA-based biomarker detection algorithm that permits the characterization of specific microbial taxa that are differentially expressed between samples belonging to two different classes.

Comprehensive comparisons with state-of-the-art biomarker discovery algorithms belonging to the class of statistical methods and the class of machine learning approaches were conducted. The obtained results were evaluated (i) statistically in terms of classification accuracy and reproducibility performance and (ii) biologically by discussing their biological relevance to the case under study and their agreement with previous studies. Experiments were conducted on two realistic datasets. The first dataset is in relation to healthy dogs and dogs diagnosed with IBD. The second dataset is a mouse model of ulcerative colitis. Experiments show that the RPCA algorithm effectively detects microbial biomarkers in both datasets. In particular, the detected biomarkers by the RPCA algorithm exhibit high accuracy in discriminating the metagenomic samples belonging to different phenotypes. More importantly, RPCA shows a high reproducibility performance when compared with the other algorithms. These findings demonstrate that (i) the concept of modeling the abundance level matrix as the sum of a low-rank matrix representing the irrelevant bacteria and a sparse matrix containing the abundances of informative bacteria, (ii) the use of RPCA to recover this sparse matrix, and (iii) the inherent multivariate nature of RPCA that handles the complex microbial interactions, were successful in finding potential metagenomic biomarkers with high reproducibility and discriminative power.

The proposed framework for assessing the reproducibility provides a simple yet effective guidance for the design and evaluation of biomarker discovery algorithms. Additionally, the proposed RPCA-based biomarker detection algorithm contributes to the identification of microbial biomarkers that might explain the differences between bacterial communities under different conditions. In addition to outperforming the current state-of-the-art metagenomic biomarker detection algorithms, the presented results reveal that RPCA is a robust and reliable tool for identifying such biomarkers.

## Reviewers’ comments

### Reviewer’s report 1: Reviewer #1: Masanori Arita, The University of Tokyo, Japan

The manuscript applies the SPCA to the metagenomic data. As far as I know, this is the first application to metagenomics although the approach has been used for other bioinformatics (gene expression analysis). The introduction part is nicely written and the comparison with other approaches is fair. Reviewer recommendations to authors:

1. The key question here concerns the benefit of using SPCA and discussion. Finding the same taxa as those reported previously was a good indication. But were the results always equal? What were the differences? Such detailed analysis are necessary in the discussion because the current manuscript only briefly compares the different approaches.

Author’s response: *The reviewer has pointed important comments. The response for these comments is divided into three parts. The first part explains the importance of the comparisons with other approaches. The second part considers the benefits of our proposed algorithm. The third part addresses the discussion of the similarities and differences between the detected markers by different approaches.*

*First part: A major bottleneck for the evaluation of biomarker discovery algorithms is the lack of “ground truth” (i.e., the true biomarkers) to objectively evaluate the performance of biomarker detection algorithms. To overcome this problem, researchers usually resort to statistical and biological evaluation of detected markers. Regarding the statistical assessment, the evaluation criteria and comparisons have to be suitably designed in order to mimic the knowledge of true markers. In particular, the evaluation metrics need to capture the features of the true biomarkers. True biomarkers are characterized by two properties. The first property is that true markers enable to distinguish samples belonging to different phenotypes. In general, this feature is measured via the classification performance of a classifier model built using only the selected biomarkers. The second feature is that true signatures tend to be robust against small variations in the training set. This feature can be assessed through the empirical estimation of the stability of a biomarker detection algorithm. Therefore, our paper includes extensive comparisons in order to provide a fairer and more accurate evaluation of the competing methods, and to reflect our confidence about the biological conclusions. For example, if an algorithm exhibits an inconsistent performance and/or poor classification performance, this puts its biological findings under question.*

*Second part: Based on the conducted comprehensive simulation experiments. The RPCA-based biomarker detection algorithm provides a high reproducibility performance with quite high discriminative accuracy irrespective of the complexity of the dataset or the number of selected biomarkers. In addition, the convex formulation of RPCA provides a natural way to incorporate prior knowledge about the biological process under study, which may lead to more accurate results.*

*Third part: The selected markers by the five competing algorithms over the two datasets have been included [see the Additional files* 1 *and* 2]. *Additionally, we included more details in the manuscript about the agreements and disagreements between the identified markers by the five algorithms over the two datasets. Please check the response for comment number 3 below for discussion regarding the canine with IBD dataset. Regarding the mouse model of UC dataset, the following paragraph was added:*

*‘The Additional file* 2, *which lists the top 10 detected markers by the five algorithms, shows that 8 out of the 10 identified markers by RPCA and LEFSe are in common. Instead of Desulfovibrio and Butyricicoccus which are identified by LEFSe, RPCA suggested Bacteroides and Lactobacillus as potential markers for UC. RPCA shows less agreement with MetaStats in which only 3 signatures are in common between them. Specifically, RPCA and MetaStats agrees on Bifidobacterium, Streptococcus, and Escherichia/Shigella as possible markers for UC. On the other hand, RPCA does not share any of its detected markers neither with the Entropy-based method nor with th BC algorithm.’*

2. In a standard viewpoint, the matrix S (perturbation) may contain noise. So the discussion to distinguish noise from the IBD key is also necessary.

Author’s response: *The optimization problem formulation for extracting the low-rank and sparse matrices provides a natural robustness against Gaussain noise. In particular, the third term in the optimization problem* (9), *which measures the error between the bacterial abundance data and the extracted low-rank and sparse matrices in the Euclidian norm, reflects the maximum likelihood estimation of the parameters (i.e.,* **L** *and* **S**) *in the presence of Gaussian noise. Therefore, we expect that the RPCA algorithm will exhibit robust performance against Gaussian noise. However, it is known that the Euclidian distance is sensitive to nonGaussian noise and outliers. In such cases, the raw bacterial abundance data may be preprocessed to mitigate the impact of such noises by using special filters such as median filters.*

3. This paper may be good as a methodology paper, but for a research, application of the MatLab code is not enough. Please enhance the biology part of this presentation more in details.

Author’s response: *We thank the reviewer for this suggestion. In response for this comment, the following comments have been added to the manuscript: ‘In order to validate the detected markers by RPCA, an independent validation experiment has been conducted. In particular, quantitative PCR (qPCR) assays targeting thirteen bacterial groups were conducted over fecal DNA samples taken from 285 healthy dogs and 172 dogs with chronic enteropathy (CE) [* 44 *]. In this experiment, the final PCR panel (i.e., Faecalibacterium, Turicibacter, E. coli, Streptococcus, Blautia, and Fusobacterium) includes five OTUs (i.e., Faecalibacterium, Turicibacter, Streptococcus, Blautia, and Fusobacterium) that are strongly suggested as potential signature for IBD by our RPCA-based algorithm. As shown in the Additional file* 1, *the RPCA is the only algorithm that suggested Faecalibacterium and Blautia as potential biomarker for IBD. In particular, Blautia was proposed as a strong driver for IBD by RPCA and three species from Blautia genera (rank 5, 9, and 18) were selected in the top 30 markers. Moreover, the Streptococcus was strongly suggested as strong potential marker for IBD by RPCA. Specifically, one species of Streptococcus was ranked second by RPCA. This agrees with Metastats which suggested two species belonging to Streptococcus genera (rank 3 and 8), and BC which suggested one Streptococcus species with rank 23 as IBD marker. On the other hand LEFSe and Entropy algorithms do not include Streptococcus in their suggested lists of IBD markers. RPCA and LEFSe are the only algorithms that suggested Turicibacter as a signature for IBD. Fusobacterium was strongly recommended as possible marker for IBD by RPCA and MetaStats (rank 12 and 5, respectively) while it is less favored by LEFSe (rank 25). This independent validation experiment demonstrates the efficiency of RPCA-based algorithm in identifying markers with high classification potential.’*

4. Instead of writing the details of the alternating based method, please inform the Matlab code and library for biology users.

Author’s response: *Done. A link for the Matlab code has been provided in the manuscript: “It is worth to mention that there are several implementations for the RPCA algorithm. In our experiments, we utilize the Matlab code for the exact ALM provided by the authors of [34], which is available at* http://perception.csl.illinois.edu/matrix-rank/sample_code.html.” *The details of the alternating based method was provided for the sake of completeness.*

5. Formula (5) was not properly printed in my printer setting. It is not recommended to separate formulas by a comma here because it looks like "FP’". Maybe inserting some space (LaTeX backslash?) is better.

Author’s response: *The authors believe that separating the three equations by comma is appropriate here.*

6. Bacterial taxonomy is not very familiar to most readers. Genus may be not enough. For example, Blautia and Ruminococcus are very close. Lactobacillus is a very large clade. Are there more general trends?

Author’s response: *For the mouse model of UC dataset, we have only the bacterial abundance data analyzed at the genus level. We do not have the original sequences to analyze the data at lower phylogenetic depth (i.e., at species level). For the canine with IBD dataset, the experiments have been conducted at the species level.*

**Quality of written English**

Acceptable

### Reviewer’s report 1: Reviewer #2: Zoltan Gaspari, Pazmany University, Budapest

The manuscript describes the application of the RPCA method for biomarker detection in metagenomic samples. The advantages of the method seem convincing and the comparative nature of the study offers important insights for those working in the field. Reviewer recommendations to authors:

1. The diversity of the samples might be one of the reason why the histograms and the performance of some methods differ substantially. Can the authors offer a measure for this?

Author’s response: *The reviewer has made an interesting comment. In fact, this is the main motivation for our work. In reality, metagenomic datasets exhibit some kind of diversity or variation among them. The platforms used to implement the experiments, the sample size, and the differences between subjects participating in the experiments are few examples of possible reasons for this variation. Therefore, it is important to develop an algorithm that provides a robust performance irrespective of the natural diversity of the samples. Indeed, measuring the diversity of the samples may provide further insights about the diversity of the bacterial compositions in the samples. In the field of ecology, researchers commonly use three measures of the biodiversity (species diversity) of the samples. These measures are the alpha diversity* (*α*−*diversity), beta diversity* (*β*−*diversity), and gamma diversity* (*γ*−*diversity). However, measuring such a diversity is out of the scope of this paper. This is because all algorithms were tested using the same samples (under the same sample diversity).*

2. Changes in the numbers of an abundant microbe relative to a rare one might be of different meaning/importance, is this factor relevant in the analysis?

Author’s response: *We thank the reviewer for raising this important point. Indeed the variation in the abundance levels might have different meanings. For some methods, this issue may lead to a serious bias problem in the sense that variables (i.e., microbes) with more abundance levels may be preferred over those with less abundance levels. For example, the Random Forest (RF) model, which is a very popular classifier and variable ranking technique is biased in favor of features with larger values* 51]. *On the other hand, our algorithm does rely on the abundance range of the variables. In fact, our RPCA-based algorithm does not require any prior knowledge about the microbial abundance profiles. Moreover, the ranks assigned by the RPCA are not correlated with the absolute and/or fold change variation in the mean values of the variables between healthy and diseased samples as it is transparent from the supplementary material added to the manuscript [see the Additional files* 1 *and* 2].

3. Kindly consider this essay about the 1:10 ration of human vs. bacterial cells: http://www.nature.com/news/ scientists-bust-myth-that-our-bodies-have-more-bacteria-than-human-cells-1.19136.

Author’s response: *Thanks. We have considered your suggestion in the manuscript. In particular, the following statement has been added to the manuscript:‘Some studies have reported that the microbes outnumber the human’s cells by a ratio of 10:1* [2], *while others limits this ratio to 1.3:1* [3].’

4. Please explain all variables in the equations (including tau, p, mu etc.)

Author’s response: *Sorry, we didn’t make it clear. Description of the variables that were not defined has been added.*

- *μ* stands for the single regularization parameter associated with the ALM formulation (after Eq. (8)).
- *τ*≥0 represents the threshold value (after Eq. (12))

5. Is a division missing from Eq. 18?

Author’s response: *Modified. Thanks.*

6. LEfSe is written with small f" on page 4 and with capital everywhere else

Author’s response: *Done. Thanks.*

**Quality of written English**

Acceptable.

## Availability of Data and Materials

The canine IBD dataset is available at https://qiita.ucsd.edu/study/description/833. The mouse model of ulcerative colitis can be found in the supplementary material of [15].

## Additional files

Additional file 1 List of top 30 detected markers by the five biomarker detection algorithms over the canine IBD dataset. (XLSX 21 kb)

Additional file 2 List of top 10 detected markers by the five biomarker detection algorithms over the mouse model of UC dataset. (XLSX 14 kb)

## Abbreviations

ALM: Augmented lagrange multiplier
BC: Binary classification
IBD: Inflammatory bowel disease
LDA: Linear discriminant analysis
NCC: Nearest centroid classifier
OTU: Operational taxonomic unit
PCA: Principal component analysis
PCP: Principal component pursuit
PLS: Partial least square
RDP: Ribosomal database project
RPCA: Robust principal component analysis
SPCA: Sparse principal component analysis
SVD: Singular value decomposition
UC: Ulcerative colitis
